# Fast recovery of house infestation with *Triatoma brasiliensis* after residual insecticide spraying in a semiarid region of Northeastern Brazil

**DOI:** 10.1371/journal.pntd.0008404

**Published:** 2020-07-20

**Authors:** Claudia Mendonça Bezerra, Silvia Ermelinda Barbosa, Rita de Cássia Moreira de Souza, Levi Ximenes Feijão, Ricardo Esteban Gürtler, Alberto Novaes Ramos, Liléia Diotaiuti

**Affiliations:** 1 Departamento de Saúde Comunitária, Faculdade de Medicina, Universidade Federal do Ceará, Fortaleza-CE, Brazil; 2 Secretaria da Saúde do Estado do Ceará, Fortaleza-CE, Brazil; 3 Grupo Triatomíneos, Instituto René Rachou, Fundação Oswaldo Cruz, Belo Horizonte, Minas Gerais, Brazil; 4 Laboratorio de Eco-Epidemiología, Departamento de Ecología, Genética y Evolución, Facultad de Ciencias Exactas y Naturales, Universidad de Buenos Aires, Ciudad Universitaria C1428EHA, Buenos Aires, Argentina; 5 Consejo Nacional de Investigaciones Científicas y Técnicas-Universidad de Buenos Aires, Instituto de Ecología, Genética y Evolución de Buenos Aires (IEGEBA), Ciudad Universitaria, C1428EHA, Buenos Aires, Argentina; Tulane University, UNITED STATES

## Abstract

The northeastern semiarid region stands out in the Brazilian context regarding the eco-epidemiology of Chagas disease, in which *Triatoma brasiliensis* is the main vector of *Trypanosoma cruzi*. Persistent house invasion threatens the relative levels of progress achieved over previous decades. We conducted an intervention trial with a five-year follow-up to assess the impacts of residual spraying with pyrethroid insecticides on house infestation with *T*. *brasiliensis* in 18 rural villages (242 houses) located in the Tauá, Ceará. House infestations were assessed by systematic manual searches for triatomines in every domestic and peridomestic habitat on five occasions. Triatomines were collected in peridomestic (57.5%), sylvatic (35.8%), and intradomiciliary (6.7%) habitats. The most important ecotopes of *T*. *brasiliensis* were containing roofing tiles, bricks or rocks (23.4% ± 9.1). Residual insecticide spraying substantially reduced baseline house infestation rates from 27.9% to 5.9% by 6 months post first spraying (MPS). The decline was substantially greater in intradomiciles (11.2% to 0.8%) than in peridomiciles (16.7% to 5%). The mean relative density of triatomines recovered its preintervention values at 14 MPS in intradomiciles, and in the main peridomestic ecotopes. The house infestation levels recorded at 14 MPS persisted thereafter despite all reinfested houses were selectively sprayed on every occasion. Overall average bug infection rates with *T*. *cruzi* in the five occasions were in intradomiciles (11.1%), peridomiciles (4.7%) and wild habitats (3.3%). In peridomicile *T*. *cruzi* infection rates decreased significantly at all stages after chemical intervention. In intradomicile, the only significant difference occurred at 20 MPS (7.7% to 30.8%). The vectorial capacity of *T*. *brasiliensis*, combined with its invasive potential from sylvatic sources and the limited effectiveness of chemical control in the harsh *caatinga* landscape, pose serious obstacles to the definite elimination of domestic transmission risks. Systematic vector surveillance supported by community participation and locally adapted environmental management measures are needed to reduce the risks of establishment of domestic transmission with *T*. *cruzi* in this region.

## Author summary

The results demonstrate the ecological potential of *Triatoma brasiliensis* in the northeastern caatinga and how this species uses the resources available in nature or in the modified environment by man. Triatomines were collected in peridomestic, sylvatic, and intradomicile habitats. House infestations were evaluated on five occasions, with spraying of infested houses. *T*. *brasiliensis* was responsible for the high rates of dispersal, infestation, colonization and *Trypanosoma cruzi* infection in all catches and environments studied here. Spraying strongly reduced the infestation, but the number of triatomines rapidly returned to previous levels. Peridomicile represents a complex set of overlapping infested sites forming a network of shelters that favor the establishment and proliferation of triatomine colonies. Molecular characterization of *T*. *cruzi* isolated from triatomines and identification of their food sources emphasize the overlap of habitats and interaction among triatomines, synanthropic reservoirs and humans, increasing the risk of parasite transmission. Therefore, it is clear the need for maintenance of vector control fully and systematically with the use of appropriate strategies where interventions should consider regional ecoepidemiologic differences and the influence of environmental, socioeconomic and cultural factors in *T*. *cruzy* transmission process into domicile environment.

## Introduction

The northeastern semiarid region stands out in the Brazilian context because of its distinctive eco-epidemiological patterns of Chagas disease, rich diversity and broad dispersion of triatomine bugs. The *Triatoma brasiliensis* Neiva, 1911 complex currently includes two subspecies (*Triatoma brasiliensis brasiliensis* Neiva, 1911 and *Triatoma brasiliensis macromelasoma* Galvão, 1956) [1] and six species: *Triatoma sherlocki* Papa et al 2002 [2], *Triatoma lenti* Sherlock & Serafim, 1967, *Triatoma juazeirensis* Costa & Felix, 2007, *Triatoma melanica* Neiva & Lent, 1941, *Triatoma bahiensis* Sherlock & Serafim, 1967 [3] and *Triatoma petrocchiae* Pinto & Barreto, 1925 [4,5]. *T*. *b*. *brasiliensis* from here on referred to as *T*. *brasiliensis*, is the most important vector of *Trypanosoma cruzi* (Chagas, 1909) [6] (Kinetoplastida, Trypanosomatidae) in the semiarid region of Brazil. It has a wide geographic distribution, high rates of infection with *T*. *cruzi*, invasive potential, and blood-feeding eclecticism. Its center of dispersion is the *caatinga* biome. As this triatomine species usually hides in rocks, it is associated with various species of bats, marsupials and rodents [7–12], and with the Xiquexique cactus *Pilosocereus gounellei* (A. Weber ex K. Schum.; Bly. ex Rowl.) in sedimentary plains [13].

Periodic residual spraying of pyrethroid insecticides is the strategy recommended for effective control of domestic triatomine species [14]. This strategy has been quite successful, especially with introduced species such as *Triatoma infestans* Klug, 1834 and *Rhodnius prolixus* Stål, 1859 [15]. However, native species with intrusive features require constant vector surveillance as new invasions and recolonization may occur early after the effects of control actions wane. Home recolonization is the restoring infestation process from triatomines active invasion from the natural environment (invaders) and/or by residual foci of triatomines after spraying. The structural complexity of the peridomicile is a key determinant of this process [10,15–19].

The occurrence of house invasion and colonization increase with the proximity between natural and artificial ecotopes and the interactions among triatomines, synanthropic animals and humans [10–12,16,20,21]. The peridomicile contains multiple structures that provide shelter to triatomines, suitable microclimatic conditions, and diverse host sources [22–25]. The combined effects of refuges for triatomines in structures in which insecticides can hardly penetrate, high temperatures, wind, rainfall and insolation reduce the duration of pyrethroids residual effects [16,21,26,27]. Because *T*. *brasiliensis* has the ability to invade and exploit different habitats (including human habitations), it has been classified as a priority species by the Brazilian Chagas Disease Vector Control Program (PCDCh) [26,28].

Measuring the effectiveness of current vector control strategies and looking into the putative mechanisms responsible for persistent or recurrent house infestation are important for achieving better outcomes and designing improved methods. The key aspects are the spatial distribution of vector populations, seasonal variations in abundance, development stage and house invasion. Knowledge on these processes may be used to predict potential risks and inform vector control programs decisions [29]. The resistance of *T*. *brasiliensis* to the pyrethroid insecticide has not been identified [30], stimulating the realization of this project, which seeks to understand the origin of infestations persistence. Here, we report the outcomes of an intervention trial with a five-year follow-up designed to assess the impacts of residual spraying with pyrethroid insecticides and subsequent selective treatments on housing unit (HU) infestation with *T*. *brasiliensis* and its infection rate with *T*. *cruzi* in 18 rural villages located in a well-defined area of Ceará.

## Materials and methods

### Ethics statements

Approved at the Chico Mendes Institute for Biodiversity Conservation, Ministry of Environment (ICMBio/MMA), through the Biodiversity Authorization and Information System (SISBIO), Protocol no. 31.693–1 and Authentication code 46619742

### Study area

**Fig 1:**
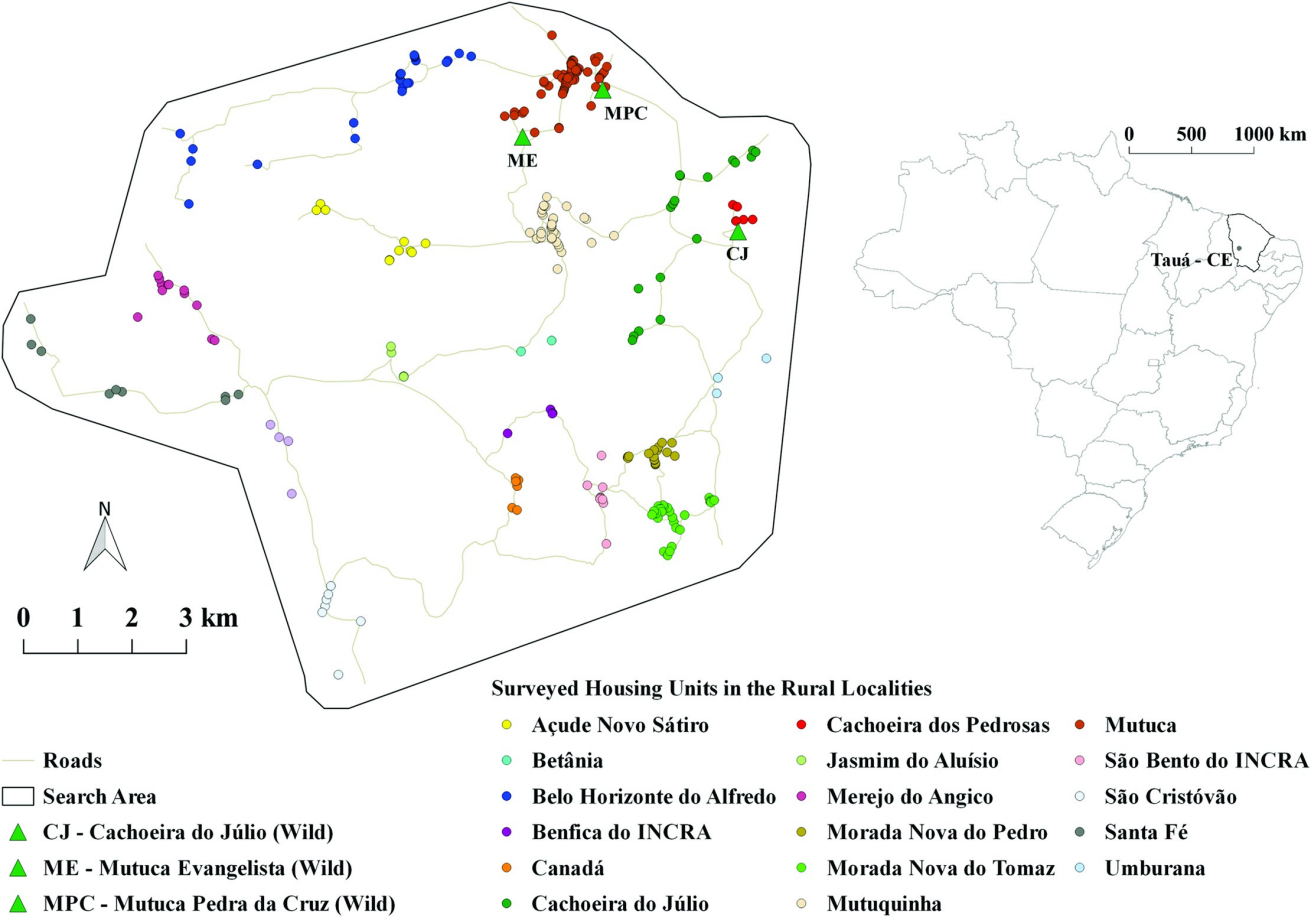
Study area in the municipality of Tauá, Ceará. **Source:** Adapted from Landsatlook viewer (landsatlook.usgs.gov) and QGis 2.14. Essen. USGS (United States Geological Survey).

This study was conducted in Tauá municipality, Ceará, Brazil (Fig 1), a traditionally endemic area for Chagas disease located in the *caatinga* biome. Tauá has 533 localities with high dispersion averages (87% - 1983–2018) and house infestation averages (23% - 1983–2018) with predominance of *T*. *brasiliensis*. For the current study we included 18 neighboring rural localities that had not been treated with insecticides for at least two years.

Tauá is located in the hinterland of Inhamuns (6°00’11”S and 40°17’34”W) at an altitude of 402.7 meters, 320 km from Fortaleza. The mean annual temperature ranges between 26°C and 28°C; the mean annual rainfall is 597.2 mm^3^ and mostly occurs from February to April. The vegetation has been severely degraded, with secondary succession and predominance of shrubby-arboreal *caatinga* [31]. Granite outcrops with cracks in rocks are part of the stony phase of the municipality. These outcrops are inhabited by reptiles, small mammals and insects, including *T*. *brasiliensis*, and constitute its primary ecotope [7].

The HU (the epidemiological unit of reference for vector control programs) comprises the main habitations (intradomicile) and all surrounding elements (peridomicile, permanent and temporary buildings, construction materials, fences, animal shelters, etc.) [32]. The intradomicile was considered as a single habitat whereas the peridomicile was divided according to the types of elements contained: chicken coop, pigsty, woodpile, stone, brick and tile, among others.

### Study design and vector surveys

This is an intervention trial with a five-year follow-up to assess the impacts of residual spraying with pyrethroid insecticides on house infestation with *T*. *brasiliensis* and its infection rates with *T*. *cruzi*.

All housing units were inspected for triatomines on five occasions: February 2009 (before first spraying), August 2009 (6 months after first spraying), April 2010 (14 months after first spraying), October 2010 (20 months after first spraying), and August 2015 (78 months after first spraying). A trained team of municipal health agents performed manual triatomines searches inside (intradomiciliar, minimum 30´) and around (peridomicile, minimum 30´) the house. And caught as many of the triatomines sighted as possible using standard procedures [32]. All field activities were supervised by personnel from the State Department of Health.

The research was explained in all HU and requested in writing to the consent of the household head, through the free and clarified term (S1 Checklist) so that we could enter and perform the work in the houses and annexes.

Following the baseline vector survey (February 2009), all housing units were sprayed with 20% CS alpha-cypermethrin (FERSOL Indústria e Comércio S/A) regardless of infestation status using routine procedures [17, 32]. Only infested housing units were sprayed in the four subsequent surveys (selective spraying) [14].

Three areas with rocky formations typical of the natural ecotopes of *T*. *brasiliensis* were selected for comparison. These areas showed different degrees of human intervention related to the distance to the closest house: Mutuca Pedra da Cruz (MPC, at 94 m) was severe intervention; Mutuca Evangelista (ME, at 565 m), was regular intervention, and Cachoeira do Júlio (CJ, at 370 m), had a low degree of intervention (Fig 1). These sylvatic habitats were manually searched for triatomines with the aid of a lantern over four consecutive nights (5:30 p.m. to 10:00 p.m.) in February 2009, August 2009, April 2010 and August 2015 by six researchers.

### Entomological indicators

The indicators [17, 32] for monitoring the risk of vector-borne transmission of Chagas disease at a house level (all numerators multiplied by 100): *infestation* (number of domestic units with presence of triatomines divided by the number of units searched); *reinfestation* (number of units with presence of triatomines divided by the number of positive units at *baseline*); *colonization* (number of units with presence of triatomine nymphs divided by the number of units with presence of triatomines (adults and nymphs); *density* (number of captured triatomines divided by the number of units with presence of triatomines), and *natural infection* (number of triatomines infected with *T*. *cruzi* divided by the number of triatomines examined). The indices can be stratified according to intradomestic and peridomestic environments.

### Laboratory tests

All captured triatomines were kept in separate tubes by time and place of capture for identification of species and developmental stage, and examination for infection with trypanosomes [33]. Triatomines were identified to species using the keys described by Lent & Wygodzinsky [34] and subsequent descriptions of the *T*. *brasiliensis* complex [1,3–5,35,36].

Two methods were used for parasitological diagnosis: 1) examination of fresh feces: these were diluted in saline solution (NaCl 0.9%), placed on a slide and cover slip, and at least 100 fields were read at 400x magnification using an optical microscope; 2) slides stained with Giemsa: 10% of the negative slides and all those that were doubtful and positive for trypanosomes were stained with methylene blue and Giemsa (Walker’s technique) for a new reading of at least 100 fields per slide under a 1,000x magnification [33]. These procedures were carried out in cooperation with the Laboratory of Entomology Dr. Tomaz Aragão, Department of Health of Ceará State (techniques 1 and 2), and the Reference Laboratory in Triatomines and Epidemiology of Chagas Disease, René Rachou Institute (IRR) at Fiocruz in Minas Gerais (technique 1).

### Geospatial analysis

The geographic coordinates of each housing unit and of the sites positive for triatomines were recorded using a 12-channel eTrex GPS (Garmin), with projection WGS89—Zone 24S. Subsequently, the points geocoded in high resolution (maximum erro 10m), generated a map embedded in the environment of the geographical information system (GIS) in accordance with the basis of Google Earth Pro softwarev.7.1.

Exploratory analysis of spatial behavior of events was based on the estimate kernel density to create a raster map where by the density was based on the number of infested points in the study region and quantity of triatomines captured. In this way, it was checked whether the events occurred at random or there were aggregations among them (hotspots), indicating the occurrence of clusters [37]. The maps were generated in the software QGisv.2.14, an open-source geographical information system (https://qgis.org/en/site/about/index.html).

### Statistical analysis

The association between infestation, ecotope and survey occasion was examined using Fisher's exact test or Pearson’s χ^2^ test, with a significance level of 5%. Analyses were performed using Stata 11.2. (StataCorp LP, 2011).

## Results

S1 Table shows the number of houses inspected for infestation and their construction patterns over the five-year follow-up. A total of 1,211 searches for triatomines were made, with an average of 242 houses inspected on any occasion. The frequency distribution of construction materials did not differ significantly over time (χ^2^ = 4.52, *df* = 4, *P* = 0.34). Brick-made houses with plastered walls accounted for 66.1% of the total, followed by brick-made houses without plaster (27.1%), clay houses without (4.3%) and with plaster (2.5%). Tile roofs were used in all houses.

**Fig 2:**
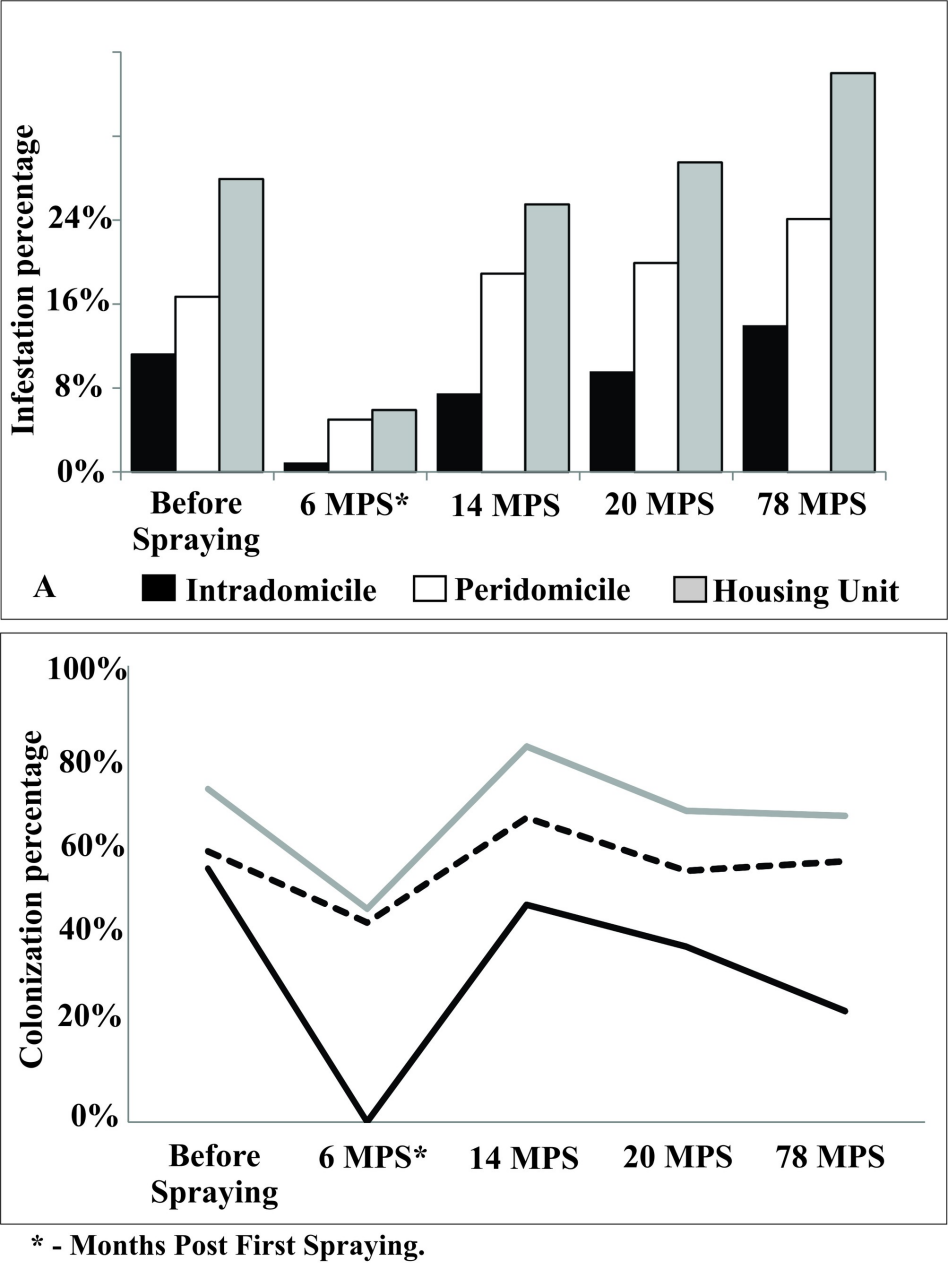
House infestation (A) and colonization (B) rates by *Triatoma brasiliensis* according to site of capture, Tauá, Ceará, 2009 to 2015.

Residual insecticide spraying highly significantly reduced baseline house infestation rates from 27.9% to 5.9% by 6 months post first spraying (MPS) (Fisher's exact test, P < 0.001) (Fig 2). The decline was more marked in the intradomicile, from 11.2% to 0.8% (Fisher's exact test, P < 0.001), than in the peridomicile, from 16.7% to 5% (Fisher's exact test, P < 0.001). At the peridomestic-site level, baseline infestation rates dropped from 10.7% to 3.9% at 6 MPS. Infestation rates at 14, 20 and 78 months post spraying did not differ significantly from baseline values in the intradomicile (χ^2^ = 5.59, *df* = 3, *P* = 0.133) or the peridomicile (χ^2^ = 0.61, *df* = 3, *P* = 0.895), or at the house level (χ^2^ = 1.81, *df* = 3, *P* = 0.612). Infestation rates increased fast between 6 and 14 MPS. The patterns observed for colonization rates were parallel to those recorded for infestation (Fig 2).

**Fig 3:**
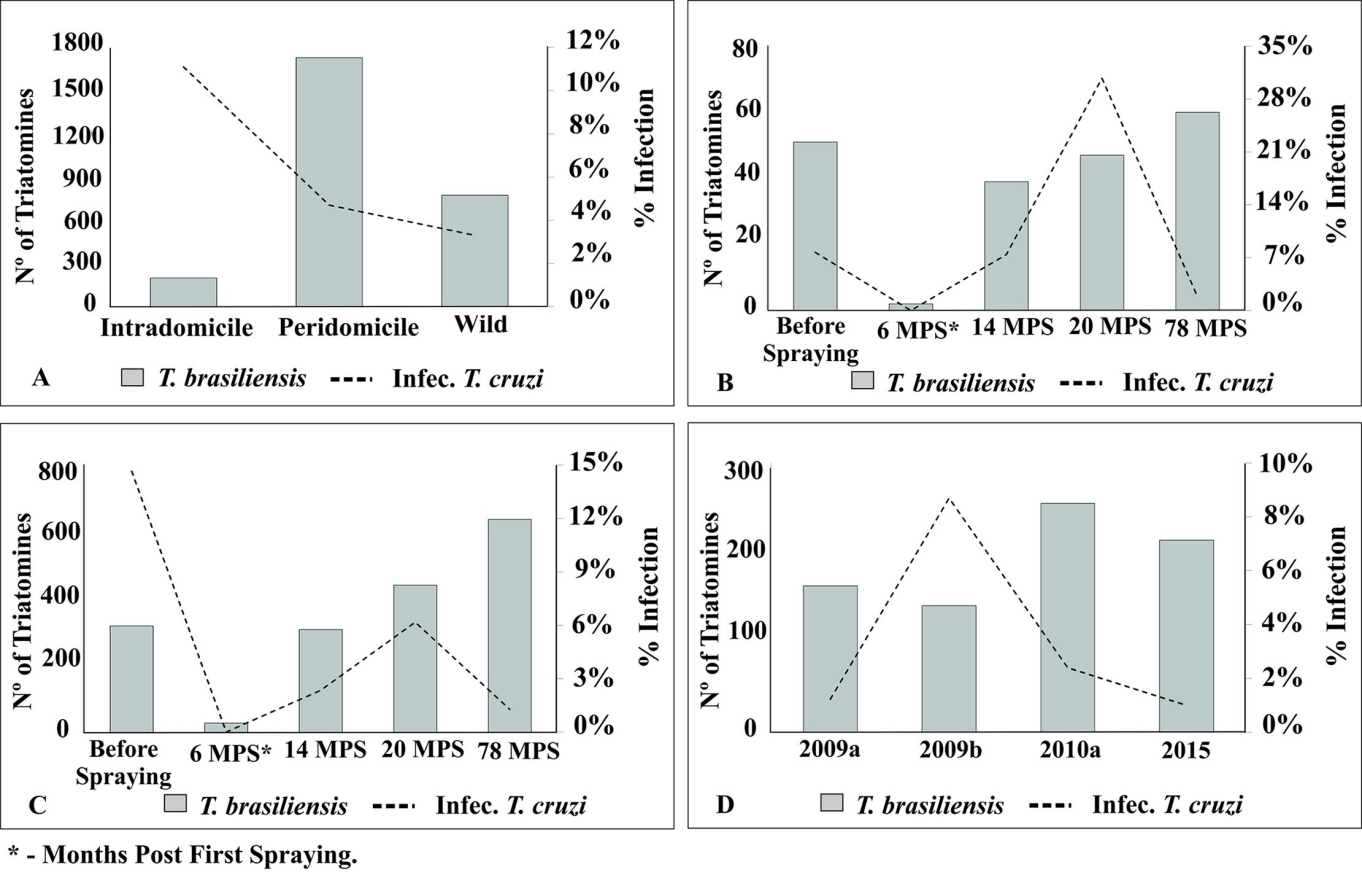
Number of *Triatoma brasiliensis* captured and rate of natural *Trypanosoma cruzi* infection, according to location and capture time in Tauá, Ceará, 2009 to 2015. **A.** General data on domicile environment (intradomicile and peridomicile) and wild environment; **B.** intradomicile; **C.** peridomicile; **D.** domicilar unit.

Of 3,005 specimens of *T*. *brasiliensis* captured throughout the five-year period, most came from peridomestic (57.5%) and sylvatic (35.8%) habitats; only 6.7% were caught in intradomiciles. However, intradomiciliary triatomines showed the topmost rates of infection with *T*. *cruzi* (11.1%), followed by those from peridomiciles (4.7%) and wild habitats (3.3%) (Fig 3, S2 Table). Intradomiciliary bug infection rates were widely variable over time both in nymphs and adults, with overall infection reaching 30.8% at 20 MPS; nymphs also displayed near-maximum levels (28.6%). In peridomestic habitats, overall infection dropped steeply after community-wide insecticide spraying (from 14.6% to 1.3%), and fluctuated at lower levels in sylvatic habitats (range, 1.2 and 8.7%). Chemical intervention performed significantly impacted *T*. *cruzi* infection between baseline and 6 MPS (13.3% to 0%) (Fisher's exact test, P <0.05). In peridomicile environment these rates were significantly lower in all periods when compared to baseline values (Fisher's exact test, P <0.001). In intradomicile, apparent reduction in infection between baseline and 6, 14 and 78 MPS was not statistically confirmed (Fisher's exact test, P> 0.05).

The development stage of *T*. *brasiliensis* in intradomiciles showed a predominance of nymphs during the first semester (both at baseline and at 14 MPS), whereas most bug captures over the second semester (at 6, 20 and 78 MPS) comprised adult triatomines. In contrast, in peridomestic habitats nymphs predominated throughout the follow-up (S2 Table and S1 Fig).

The preintervention frequency distribution of infested ecotopes showed top values (45.7%) in roofing tiles, bricks or rocks, followed by chicken coops (30.4%), other structures (13%), and wooden habitats (8.7%) (Table 1). This ranking approximately held after interventions, except for a relative decrease in the infestation of chicken coops and an increase in wooden habitats.

**Table 1:** Frequency of peridomicile ecotypes infested in Tauá, Ceará, 2009 to 2015.

Ecotope Type	Before spraying n (%)	6 MPS* n (%)	14 MPS n (%)	20 MPS n (%)	78 MPS n (%)	Total n (%)	Median	Media ± standard deviation
**Roofing tiles, bricks and stones**	21 (45,7)	7 (43,8)	26 (47,3)	30 (51,7)	33 (50,8)	117 (48,8)	26	23,4 ± 9,1
**Chicken coop**	14 (30,4)	2 (12,5)	10 (18,2)	8 (13,8)	3 (4,6)	37 (15,4)	8	7,4 ± 4,5
**Firewood**	4 (8,7)	3 (18,8)	9 (16,4)	6 (10,3)	16 (24,6)	38 (15,8)	6	7,6 ± 4,7
**Pigsty**	1 (2,2)	2 (12,5)	3 (5,5)	1 (1,7)	2 (3,1)	9 (3,8)	2	1,8 ± 0,7
**Others**	6 (13)	2 (12,5)	7 (12,7)	13 (22,4)	11 (16,9)	39 (16,3)	7	7,8 ± 3,9
**Total**	**46 (100)**	**16 (100)**	**55 (100)**	**58 (100)**	**65 (100)**	**240 (100)**	**55**	**48 ± 17,1**

* Months Post First Spraying. n = absolute number

**Fig 4:**
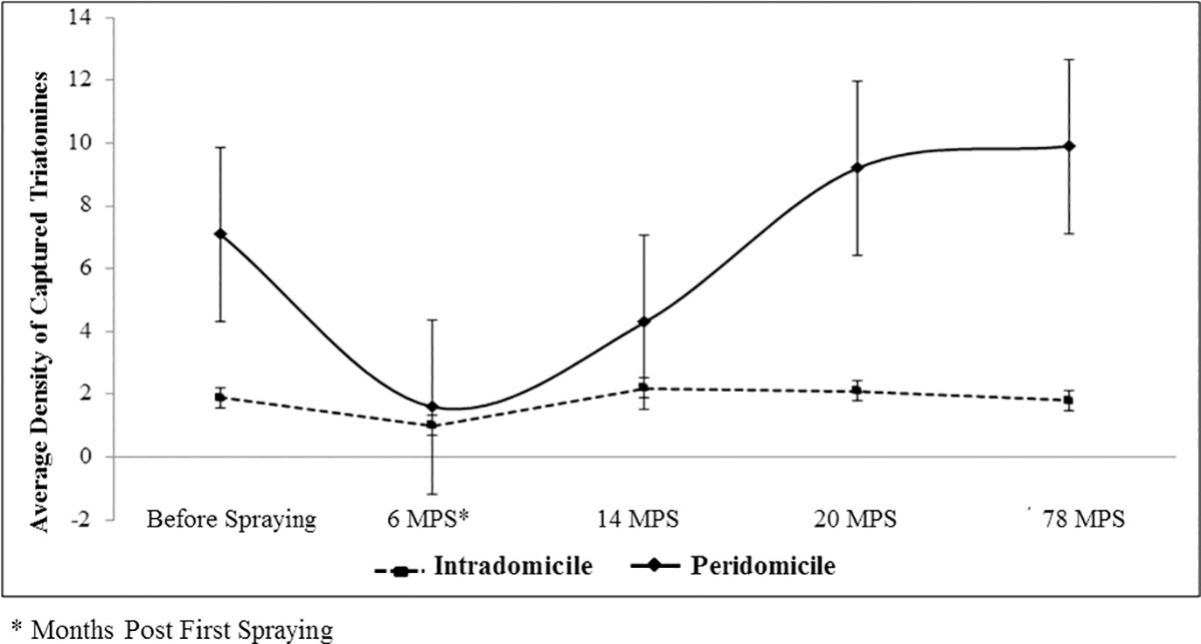
Average density of captured triatomines by infested ecotopes, according to the period and place of capture in Tauá, Ceará, 2009 to 2015.

Following the community-wide spraying campaign, the mean relative density of triatomines recovered its preintervention values at a different pace according to type of habitat (Fig 4). Baseline densities were recovered at 14 MPS in intradomiciles, in ecotopes including roofing tiles, bricks or rocks, and in chicken coops.

**Fig 5:**
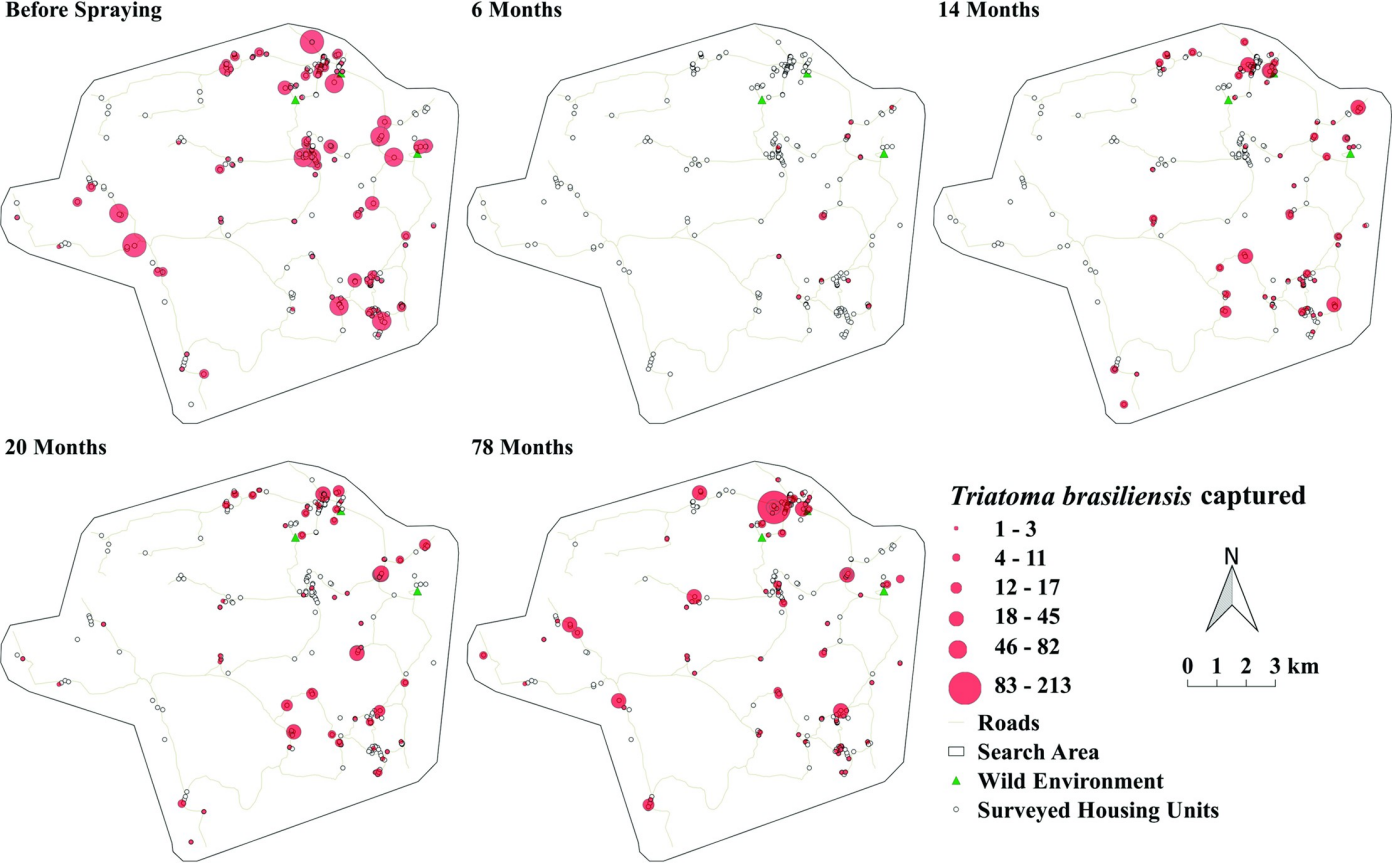
Relative abundance of *Triatoma brasiliensis* captured in Tauá, Ceará, 2009 to 2015. **Source:** Adapted from Landsatlook viewer (landsatlook.usgs.gov) and QGis 2.14. Essen.

Fig 5 shows the spatial distribution of housing unit infestation was dispersed through the municipality before interventions, with greater bug catches on the eastern section where house density was greater. Incipient infestations were recorded 6 months post spraying, and by 14 MPS it reached all district sections and remained so in subsequent surveys despite all the infested housing unit were selectively sprayed on every survey. The median of the distances of the positive peridomicile ecotopes from the households ranged from 9m (78 months) to 13m (14 months).

Between the first and fourth assessment, 28 ecotopes were reinfested, with the following frequency: two at six months, nine at 14 months, seven at 20 months and 10 at 78 months. The intradomicile was the only ecotope that was reinfested on all occasions, accounting for 60.4% (17/28) of the times. Reinfestation occurred only once in 59% (10/17) of the intradomiciles, twice in two domiciles, and one domicile was reinfested in three of the four evaluations. In the peridomicile environment, roofing tiles, stones and bricks (18%) and wood (10.8%) were the most important ecotopes (Table 2).

**Table 2:** Ecotopes reinfested by *Triatoma brasiliensis* in four evaluations carried out in Tauá, Ceará, 2009–2015.

Evaluation	N°. of reinfested ecotopes n (%)						
	Intradomicile n (%)	Roofing tiles, bricks and stones n (%)	Chicken coop n (%)	Firewood n (%)	Pigsty n (%)	Others n (%)	Total n
6 MPS*	1 (50)	-	1 (50)	-	-	-	2
**14 MPS**	5 (55,6)	2 (22,2)	1 (11,1)	1 (11,1)	-	-	9
**20 MPS**	5 (71,4)	2 (28,6)	-	-	-	-	7
**78 MPS**	6 (60)	1 (10)	-	2 (20)	-	1 (10)	10
**Total**	**17 (60,4)**	**5 (18)**	**2 (7,2)**	**3 (10,8)**	-	**1 (3,6)**	**28**

*—Months Post First Spraying. n = absolute number

## Discussion

The present study reinforces the complexity of factors involved in the process of reinfestation in the semiarid region of northeastern Brazil. In the scenario of Tauá, there was evidence of dispersal and multicausality, based on the longitudinal analysis of the main indicators of the entomological surveillance of Chagas disease. There was a significant association between abundance of triatomines and spatial analysis, with the existence of patterns of infestation and reinfestation by triatomines. Triatomines manage to return to the intradomicile and peridomicile environments very early on, with insects shifting between artificial and natural ecotopes.

PCDCh advocates cycles of active search of triatomines in areas with high-risk household transmission of Chagas disease [38]. Because of structural and operational limitations, scheduled searches are not always performed, hence native triatomine populations can reestablish [16,21,28]. In fact, rates of infestation, colonization and infection by *T*. *cruzi* in the domestic environment are expected to reduce over time if the risk areas are subjected to repeated treatments with residual insecticides [39,40]. The rates described in the present study show that *T*. *brasiliensis* reached similar levels to those found in the period before spraying 14 MPS, as described for Diotaiuti (*et al*. 2000) [16]. In Argentina (Gürtler *et al*. 2005) [40], failure in entomological surveillance in areas with *T*. *infestans* led to a recovery of this insect population 2–3 years after spraying.

In the peridomicile, the existence of ecotopes in complex conditions is a determinant factor for persistent colonization by triatomines, which take refuge in locations inaccessible to agents responsible for combating endemic diseases. In addition, one needs to recognize the limitation of the capture technique regularly used to detect the presence of triatomines. Such limitation may result in false negative data for infestation [41]. Therefore, it is very likely that infestation is much higher than the rates shown by the indicators reported in this study.

**Fig 6:**
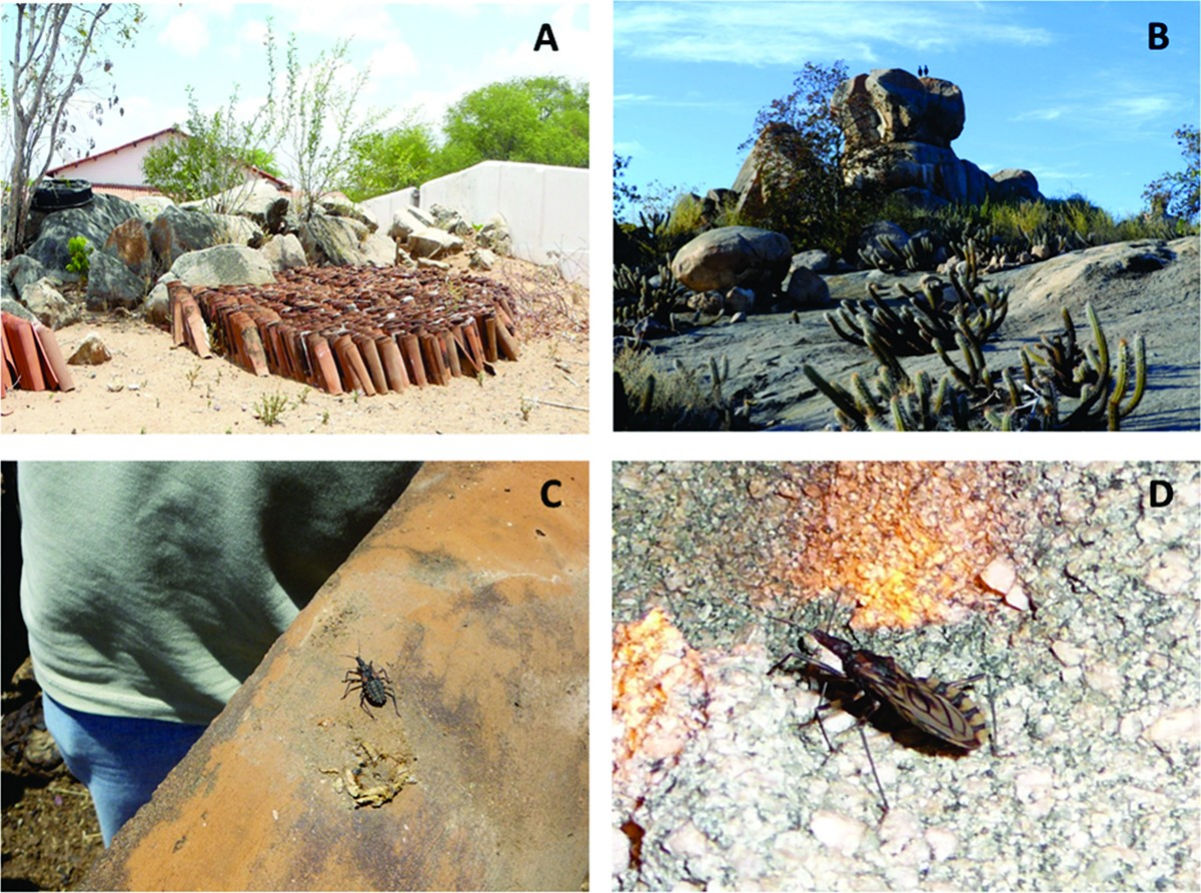
Ecotopes peridomicile and wild important for maintenance of triatomine in Tauá, Ceará. **A.** roofing tiles and rocks in peridomicile; **B.** rocks in wild environment; **C.** and **D.** triatomine found in the ecotopes, respectively.

Throughout the captures, nymphs and adults were found inside the intradomicile. Although density was low, it clearly signals the process of colonization in this environment. The presence of these adults may be due to the occasional invasion of insects that originate in the peridomicile or the wild environment, mainly in the driest periods of the year (Fig 6). The house dwellers report that adult insects frequently fly into the households, which does not exclude the possibility of passive dispersal, as the peridomicile ecotopes are near the households. Considering the long period (319 days) for development of the evolutionary cycle of *T*. *brasiliensis* [42], the fact that insects at different stadium of development were found in the peridomicile, in all sampled periods, is indicative of insects that remained even after chemical control, in addition to those coming from the wild environment [10,18,23,24,43–48].

Peridomicile ecological network, composed of artificial ecotopes, creates favorable conditions for proliferation of triatomine colonies, which vary in time and space, as they are renewed and used by domestic animals. The results of this study show that roofing tiles, bricks and stones and wood served as stable ecotopes, leading to an increase of infestation and density of triatomines throughout the period, providing shelter and availability of food sources (Fig 6A and 6C). The active and passive dispersal of these insects between ecotopes is favored when these structures are built or modified between a chemical intervention and another [10,16,18,21,49]. In the same region, a study previously published by Bezerra *et al*., (2018) [50] indicates the intense dynamics of *T*. *brasiliensis* in intradomicile, peridomicile and wild environments, through the identification of DNA from 20 different species used as food sources (rodents, goats, oxen, and cats), including marsupial DNA, pigs, and horses in intradomicile *T*. *brasiliensis*.

The spatial distribution of triatominae reveals that the process of infestation is dispersed, with small foci in the 1^st^ evaluation (6 MPS). Although these housing unit were sprayed again, there were ecotopes infested with a big number of colonies after the 2^nd^ evaluation. This result should be taken into consideration for the purpose of entomological surveillance of Chagas disease, because the first chemical intervention covered all existing houses unit in the localities, both in the intradomicile and the peridomicile, resulting in a significant impact on infestation. The same result was not found in subsequent evaluations, because the housing unit had been selectively sprayed, according to the current national guidelines [14]. Evidently, the methodology of traditional spraying has very limited effects on the control of *T*. *brasiliensis*, and a more detailed assessment is required in the quest for appropriate methodologies in this context. The present results refer to the limited effectiveness of residual spraying for control of triatomines observed in Argentina’s Gran Chaco. Gurevitz *et al*, (2013) [51] underscored the need for regular applications of insecticides, complemented by policies to improve housing units with a view to reducing the availability of habitats for insects. The application of higher concentrations of pyrethroid [52] also caused a greater reduction of infestation and time of reinfestation in this region, and it could be an alternative for control of *T*. *brasiliensis*, or even an increase in the spraying cover (as in the protocol of the first step in this work). However, in addition to high costs of the insecticide, there would be greater environmental contamination. New alternatives are desirable, e.g., greater sensitivity in infestation detection, either by manual capture or use of traps; use of equipment that allows better distribution of the insecticide within complex and ragged structures. All of them should be accompanied by excellent technical supervision to ensure the quality of the actions performed. Also, it is essential that the use of chemicals be accompanied by increased social participation, with wide dissemination of information, stages of vector control, and possibilities of people’s participation in decisions that will enable the transformation of reality in an environment that hinders the colonization by vectors of Chagas disease.

The finding that there were *T*. *brasiliensis* specimens infected by *T*. *cruzi* in virtually all environments and periods of capture reinforces the complexity and interconnection of cycles of the parasite in the region, where the peridomicile plays a fundamental role, keeping the cycle of *T*. *cruzi* very close to people, increasing the risk of transmission of *T*. *cruzi*. Contiguity between natural and artificial ecotopes and overlapping habitats are contributing factors, as they facilitate the interaction among triatomines, synanthropic reservoirs and humans, [10,49,50,53], as observed by Bezerra *et al*., (2014) [54] through the infection of 38% (20/53) in dogs and 6% (2/34) in pigs, or by the occurrence of TcI, TcII and TcIII in intradomicile environment, and wild Bezerra *et al*., (2018) [50].

Classical transmission of Chagas disease is linked to poverty because of the association between vectors and precarious housing [55,56]. Results reported in this study show that in only 66% of the houses the walls are made of bricks and covered with plaster. The type and condition of the walls determine the persistence of pyrethroid insecticides [57] sprayed on the surfaces.

The assessment of susceptibility of *T*. *brasiliensis* to deltamethrin performed by Pessoa *et al*., (2015) [30] showed that the domestic populations of this species are not resistant to pyrethroid insecticides; hence they can be controlled by means of residual spraying. Thus, persistence of infestation by *T*. *brasiliensis* in this area is due to possible operational failures that need to be evaluated, and also to ecobehavioral characteristics of the species, including proximity of the wild environment to human dwellings. Therefore, the results require that permanent, thorough and regular entomological surveillance be maintained in the region, with broad participation of the community, while considering environmental, socioeconomic and cultural factors in the ecoepidemiological context of maintenance and transmission of *T*. *cruzi* in the domestic environment (57). Importantly, surveillance activities should be integrated with health care procedures within the target territories to broaden the possibilities of prevention (57).

Thus, our results indicate that vector control proposed in Brazil for species such as *T*. *brasiliensis*, with widespread occurrence in wild ecotopes and frequent home colonies [58] needs to be revised. Human population in Caatinga northeastern lives in an intimate and persistent relationship with triatomines, domestic animals, synanthropic and wild animals infected with *T*. *cruzi* [23,43,49,54], reproducing outdated methods of agriculture production with damage to nature. It is known that preservation of ecosystems helps to maintain their diversity and with this the prevalence of infectious diseases is usually reduced [59,60]. Health authorities recognize the need to integrate health, environmental, vector and reservoir surveillance actions with epidemiological surveillance actions [58]. However, the work routine, at most, is limited to traditional capture and spraying actions. In other words, it is urgent to include in debate on Chagas disease entomological surveillance the sustainable use of natural resources. And so, look for strategies for regional biodiversity conservation and maintenance of essential ecological services for maintaining populations in rural areas [61]. Only then intended integration among health, environment and sustainable development will be possible [58], including health and environmental surveillance actions, vectors and reservoirs in epidemiological surveillance strategies. This integrated view makes it possible to indicate the risk of sustained vector transmission, timely identified and with a sustainable action plan and cost-benefit ratio.

## Supporting information

S1 Checklist Informed Consent Form. (PDF)

S1 Table Construction material used on houses walls surveyed in the study area in Tauá, Ceará, 2009 to 2015. (XLSX)

S2 Table *Triatoma brasiliensis* captured, examined, positive and rate of natural infection by *Trypanosoma cruzi* according to place and stage of capture, Tauá, Ceará 2009 to 2015. (XLSX)

S1 Fig Development stage of *Triatoma brasiliensis* populations captured in Tauá, Ceará, 2009 to 2015. **A.** *T*. *brasiliensis* captured in the intradomicile; **B.** *T*. *brasiliensis* captured in the peridomicile.
(TIF)
