# Supplementary material for: Fast recovery of house infestation with Triatoma brasiliensis after residual insecticide spraying in a semiarid region of Northeastern Brazil
Source: PLoS Negl Trop Dis. 2020 Jul 20;14(7):e0008404. doi: 10.1371/journal.pntd.0008404 (PMC7371158; doi:10.1371/journal.pntd.0008404)
Supplement: S1 Checklist — (PDF) [file pntd.0008404.s001.pdf]

TERMO DE CONSENTIMENTO LIVRE E ESCLARECIDO

A Secretaria da Saúde do Estado do Ceará, em parceria com o Centro de Pesquisa René Rachou, da Fundação Oswaldo Cruz (CPqRR/FIOCRUZ-MS), e a Secretaria da Saúde do município de Tauá, está realizando um trabalho em algumas localidades do Distrito Carrapateiras, que tem por objetivo descrever o padrão espaço-temporal da reinfestação pelo *Triatoma brasiliensis* na área de caatinga do município de Tauá (CE), após borrifação com inseticida residual, buscando levantar informações que possam ajudar ao programa de controle da doença de Chagas.

Para realizar a pesquisa será necessário procurar esse inseto, que popularmente é conhecido como barbeiro, bicudo, chupão, nas casas e nos seus anexos. Vimos solicitar ao senhor(a) a permissão para entrarmos em sua residência e procurar pelo referido inseto em todas as dependências, bem como, no quintal, galinheiros, chiqueiros, cerca e qualquer outro ambiente que possa abrigá-lo.

Salientamos que se encontrarmos o barbeiro, mediante sua autorização, a casa e os anexos serão borrifados com inseticida de efeito residual, chamado de Alfacipermetrina SC 20%. Este produto não costuma causar reação alérgica em pessoas ou animais.

A participação é importante para que as autoridades de Saúde Pública saibam onde ainda existe a doença de Chagas, a participação é voluntária e sem pagamento.

Para poder realizar o trabalho é necessária sua autorização, assinando ou colocando o polegar abaixo.

Tendo alguma dúvida, pode perguntar ao agente de endemias, ou de saúde, que está realizando a pesquisa em sua casa, ou ao pesquisador responsável abaixo descrito, em qualquer momento, para esclarecê-la.

Nome do Pesquisador Responsável: Claudia Mendonça Bezerra.
Endereço: Rua Nogueira Acioli, 150, apartamento 402 – Centro / Fortaleza – Ceará.
CEP: 60.110-140
Telefones: (85) 3251-1631 (residência); 3101-5442 (trabalho); 9601-3939 (celular).

Decidindo participar desta pesquisa, leia atentamente o termo acima e assine abaixo. Agradecemos a colaboração, mas ninguém é obrigado a participar. Salientamos que serão disponibilizados seus dados na pesquisa sempre que solicitado.

Data _____/____/_____

Eu, _____________________________________, concordo em participar da pesquisa acima descrita, estando ciente dos esclarecimentos que me foram dados.

DIGITAL

____
Assinatura do responsável
