# Supplementary figures and images for: Fast recovery of house infestation with Triatoma brasiliensis after residual insecticide spraying in a semiarid region of Northeastern Brazil
Source: PLoS Negl Trop Dis. 2020 Jul 20;14(7):e0008404. doi: 10.1371/journal.pntd.0008404 (PMC7371158; doi:10.1371/journal.pntd.0008404)

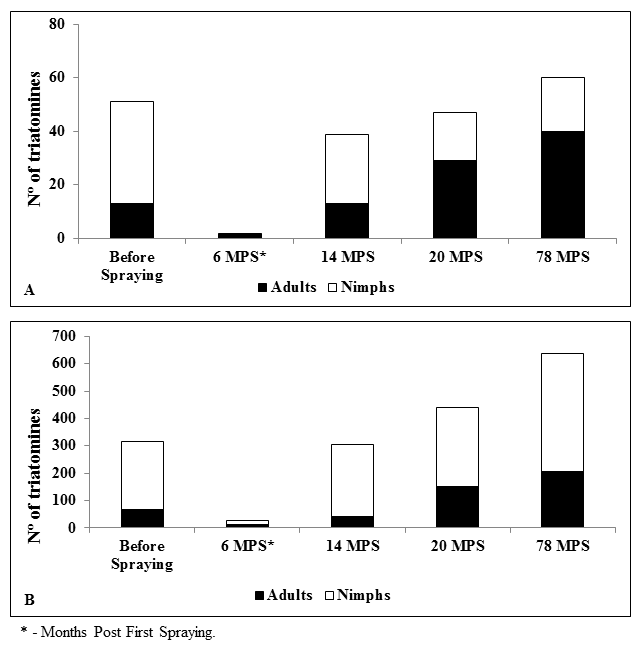

Supplement: S1 Fig — A. T. brasiliensis captured in the intradomicile; B. T. brasiliensis captured in the peridomicile. (TIF) [file pntd.0008404.s004.tif]
